# A machine-learning regional clustering approach to understand ventilator-induced lung injury: a proof-of-concept experimental study

**DOI:** 10.1186/s40635-024-00641-8

**Published:** 2024-07-02

**Authors:** Pablo Cruces, Jaime Retamal, Andrés Damián, Graciela Lago, Fernanda Blasina, Vanessa Oviedo, Tania Medina, Agustín Pérez, Lucía Vaamonde, Rosina Dapueto, Sebastian González-Dambrauskas, Alberto Serra, Nicolas Monteverde-Fernandez, Mauro Namías, Javier Martínez, Daniel E. Hurtado

**Affiliations:** 1 Facultad de Ciencias de la Vida, Universidad Andres Bello, Santiago, Chile; 2 Unidad de Paciente Crítico Pediátrico, Hospital El Carmen Dr. Luis Valentín Ferrada, Santiago, Chile; 3 Departamento de Medicina Intensiva, Pontificia Universidad Católica de Chile, Santiago, Chile; 4 Centro Uruguayo de Imagenología Molecular (CUDIM), Montevideo, Uruguay; 5 Unidad Académica de Medicina Nuclear e Imagenología Molecular, Hospital de Clínicas, Universidad de la República, Montevideo, Uruguay; 6 Academia Nacional de Medicina, Montevideo, Uruguay; 7 Unidad Académica de Neonatología, Facultad de Medicina, Universidad de la República, Montevideo, Uruguay; 8 Department of Structural and Geotechnical Engineering, School of Engineering, Pontificia Universidad Católica de Chile, Santiago, Chile; 9 Institute for Biological and Medical Engineering, Schools of Engineering, Medicine and Biological Sciences, Pontificia Universidad Católica de Chile, Santiago, Chile; 10 Departamento de Pediatría y Unidad de Cuidados Intensivos de Niños del Centro Hospitalario Pereira Rossell, Facultad de Medicina, Universidad de la República, Montevideo, Uruguay; 11 Red Colaborativa Pediátrica de Latinoamérica (LARed Network), Montevideo, Uruguay; 12 Centro Asistencial del Sindicato Médico del Uruguay (CASMU), Montevideo, Uruguay; 13 Cuidados Intensivos Pediátricos y Neonatales (CINP), Medica Uruguaya, Montevideo, Uruguay; 14 Fundación Centro Diagnóstico Nuclear, Buenos Aires, Argentina; 15 Hospital Central de las Fuerzas Armadas (HCFFAA), Montevideo, Uruguay

**Keywords:** Mechanical ventilation, Ventilator-induced lung injury, Lung strain, Computed tomography, Diagnostic imaging

## Abstract

**Background:**

The spatiotemporal progression and patterns of tissue deformation in ventilator-induced lung injury (VILI) remain understudied. Our aim was to identify lung clusters based on their regional mechanical behavior over space and time in lungs subjected to VILI using machine-learning techniques.

**Results:**

Ten anesthetized pigs (27 ± 2 kg) were studied. Eight subjects were analyzed. End-inspiratory and end-expiratory lung computed tomography scans were performed at the beginning and after 12 h of one-hit VILI model. Regional image-based biomechanical analysis was used to determine end-expiratory aeration, tidal recruitment, and volumetric strain for both early and late stages. Clustering analysis was performed using principal component analysis and K-Means algorithms. We identified three different clusters of lung tissue: Stable, Recruitable Unstable, and Non-Recruitable Unstable. End-expiratory aeration, tidal recruitment, and volumetric strain were significantly different between clusters at early stage. At late stage, we found a step loss of end-expiratory aeration among clusters, lowest in Stable, followed by Unstable Recruitable, and highest in the Unstable Non-Recruitable cluster. Volumetric strain remaining unchanged in the Stable cluster, with slight increases in the Recruitable cluster, and strong reduction in the Unstable Non-Recruitable cluster.

**Conclusions:**

VILI is a regional and dynamic phenomenon. Using unbiased machine-learning techniques we can identify the coexistence of three functional lung tissue compartments with different spatiotemporal regional biomechanical behavior.

**Supplementary Information:**

The online version contains supplementary material available at 10.1186/s40635-024-00641-8.

## Background

Ventilator-induced lung injury (VILI) is a condition of mechanical origin, with local and distant biological consequences triggered by high energy dissipation that occurs primarily on the lungs, where a high strain and stress imposed by mechanical ventilation (MV) injures the lungs [1]. Unfortunately, global measurements, such as tidal volume (Vt) or plateau pressure (Pplat), can be inaccurate in estimating strain and stress, mainly because of the large interindividual variability in end-expiratory lung volume and chest wall elastance, respectively [2], but also because of the heterogeneity of lung parenchyma affectation.

Protti et al. have identified a critical threshold of global strain capable of inducing VILI in healthy lungs if are sustained over time [3]. In a previous contribution, our group described a regional colocalization between lung inflammation and high strain during an experimental model of VILI [4]. However, the critical threshold at regional level remains unclear, where phenomena, such as regional strain, heterogeneity and tidal recruitment have been identified as potential stress raisers [5, 6]. Moreover, their topographic distribution over time and regional interactions are currently unknown. Theoretically, pathophysiology of VILI starts with alveolar instability, followed by cyclic opening and closing events, and culminating in alveolar flooding and collapse [3]. Tidal recruitment may exacerbate lung injury by subjecting alveolar walls to shear stress during tidal expansion and causing overstretching of neighboring alveolar structures. Airspaces collapse, whether at the alveolar or regional level, can amplify alveolar-wall stress in the adjacent aerated lung parenchyma, increasing them by up to four times compared to the overall tension experienced during tidal ventilation [7]. This phenomenon, referred to as alveolar interdependence, can foster both tidal overdistension and tidal recruitment in a heterogeneous lung, perpetuating a detrimental cycle.

Based on the preceding arguments, we hypothesized that we could recognize regional VILI mechanisms and to describe their temporal and spatial behave during a 12-h model of VILI.

## Methods

The study was approved by the Animal Ethics Committees in Universidad de la República (ID 070153-000775-17) and Universidad Andrés Bello (ID 021/2018). The protocol was designed following the National Institute of Health’s guidelines (NIH).

### Acute lung injury animal model

We studied 10 piglets (Sus scrofa domestica), aged 2–3 months, weighing 25–30 kg. The animals were anesthetized with an intramuscular injection of xylazine (2 mg/kg) and ketamine (20 mg/kg), followed by a continuous intravenous infusion of ketamine (30 mg/kg/h), fentanyl (0.5–1.0 μg/kg/h), midazolam (0.1 mg/kg/h), and rocuronium (0.3 mg/kg/h). Ringer’s lactate 30 ml/kg/h was infused IV during the first hour. Then it was decreased to 10 ml/kg/h until the end of the experiment.

The animals were placed in supine position and, after tracheal intubation, were mechanically ventilated (Hamilton-G5, Hamilton Medical, Switzerland) in a volume-controlled ventilation (VCV) mode using the following settings: Vt 8 ml/kg, respiratory rate (RR) 30/min, positive end-expiratory pressure (PEEP) 5 cmH_2_O, inspiratory to expiratory ratio (I:E) 1:2, and oxygen inspired fraction (FIO_2_) of 1. The animals were continuously monitored with electrocardiogram (ECG) and pulse oximetry. Also, invasive systemic arterial pressure, cardiac output, global end-diastolic volume (GEDV) and extravascular lung water (EVLW) were monitored through a femoral arterial catheter using the PICCO system (PV2015L20, Pulsion, Munich, Germany). A bladder catheter was surgically inserted to measure urine output per hour. Ventilator parameters [peak inspiratory pressure (PIP), Pplat, total PEEP (tPEEP), driving pressure (ΔP = Pplat–tPEEP), mean airway pressure, and respiratory system compliance (C_RS_, ml·cmH_2_O^−1^·kg^−1^)] were assessed. Gas exchange analysis, arterial lactate, and hemoglobin were measured using a bedside blood analyzer (iSTAT^®^-1 immunoready). Central venous hemoglobin oxygen saturation (ScvO_2_) was assessed in blood samples from the superior cava vein catheter using the iSTAT^®^ analyzer. Data were collected at (1) baseline: before lung injury; (2) early stage: at the beginning of the injurious MV; (3) late stage: after 12 h of injurious MV.

After the initial preparation, the pigs were stabilized for 30 min, and baseline measurements were recorded. The one-hit VILI model [3] was induced using the following ventilator parameters: Vt 30 ml/kg, zero end-expiratory pressure (ZEEP), RR 20/min, and I:E ratio 1:2. This setting was applied for 12 h or until decease, whichever occurred first.

### Lung image acquisition, processing, and regional analysis

Computed tomography (CT) scans were acquired with a Siemens ECAT EXACT HR + scanner at early and late stages of the experimental protocol. The CT acquisition settings were the following: 120 kVp, 173–575 mAs, pitch 1, slice thickness 0.75 mm, collimation 64 × 0.625 mm, and a gantry rotation time of 0.5 s. End-inspiratory (EI) and end-expiratory (EE) images were obtained during 5-s-long inspiratory and expiratory holds, respectively.

The lung domains were segmented from CT images using the active-contour method implemented in the ITK-Snap software (Philadelphia, USA). Two masks for EE and EI images were generated during the segmentation. The first was a whole-lung mask, considering the entire lung domain. The second mask considered only the aerated lung, including poorly aerated, normal, and hyperaerated compartments, according to predefined ranges of Hounsfield Unit (HU) values [8], and neglected regions with HU values in the non-aerated range. The difference between both masks defines the non-aerated compartment.

Image-based biomechanical analysis for the construction of regional strain and aeration maps was performed following the approach introduced by our group in previous publications [9, 10]. In brief, the NiftyReg library was employed to perform image registration between aerated-lung masks of EE and EI to obtain the displacements between the expiratory and inspiratory states of the lung [11]. A 3D tetrahedral finite-element mesh was created from the aerated-lung mask at EI for each lung of all subjects. The displacement of the mesh from EE to EI allowed for the calculation of regional volumetric strain. The finite-element meshes were divided isovolumetrically in 10 segments along the basal-to-apical direction, 10 segments along the ventral-to-dorsal direction, and 3 segments along the right-to-left direction. The intersection of these divisions along three perpendicular directions give rise to a set of regions of interest (ROI) which we further consider for biomechanical analysis. We exclude ROIs that were deemed too small for analysis. These ROIs remained consistent across all subjects, ensuring comparability of lung regions across the study.

For each ROI, the following ROI-averaged quantities were computed:

- **End-expiratory aeration**, defined as the content of gas in the ROI at end of expiration. This is directly related to the intensity as end-expiratory gas fraction (GF_EE_) = -*I*/1000, where $I$ is the ROI intensity in HU.
- **End-inspiratory aeration**, defined as the content of gas the ROI at the end of inspiration. This is directly related to the intensity as end-inspiratory gas fraction (GF_EI_) = *-I*/1000 $,$ where $I$ is the ROI intensity in HU.
- **Tidal recruitment, also known as** the delta gas fraction in the ROI, defined as $\Delta \text{gas fraction}={\text{GF}}_{\text{EI}}-{\text{GF}}_{\text{EE}}$. A value of $\Delta \text{GF}$ = 0.1 is equivalent of a difference of 100 HU [12].
- **Volumetric strain,** defined as the weighted average of volumetric strain among the finite elements contained in the ROI [4, 13]. Volumetric strain can be computed as the ratio of the volume variation of a particular ROI between inspiratory and expiratory condition. Vol strain = (inspiratory volume − expiratory volume)/expiratory volume.

### Clustering analysis

**Fig. 1:**
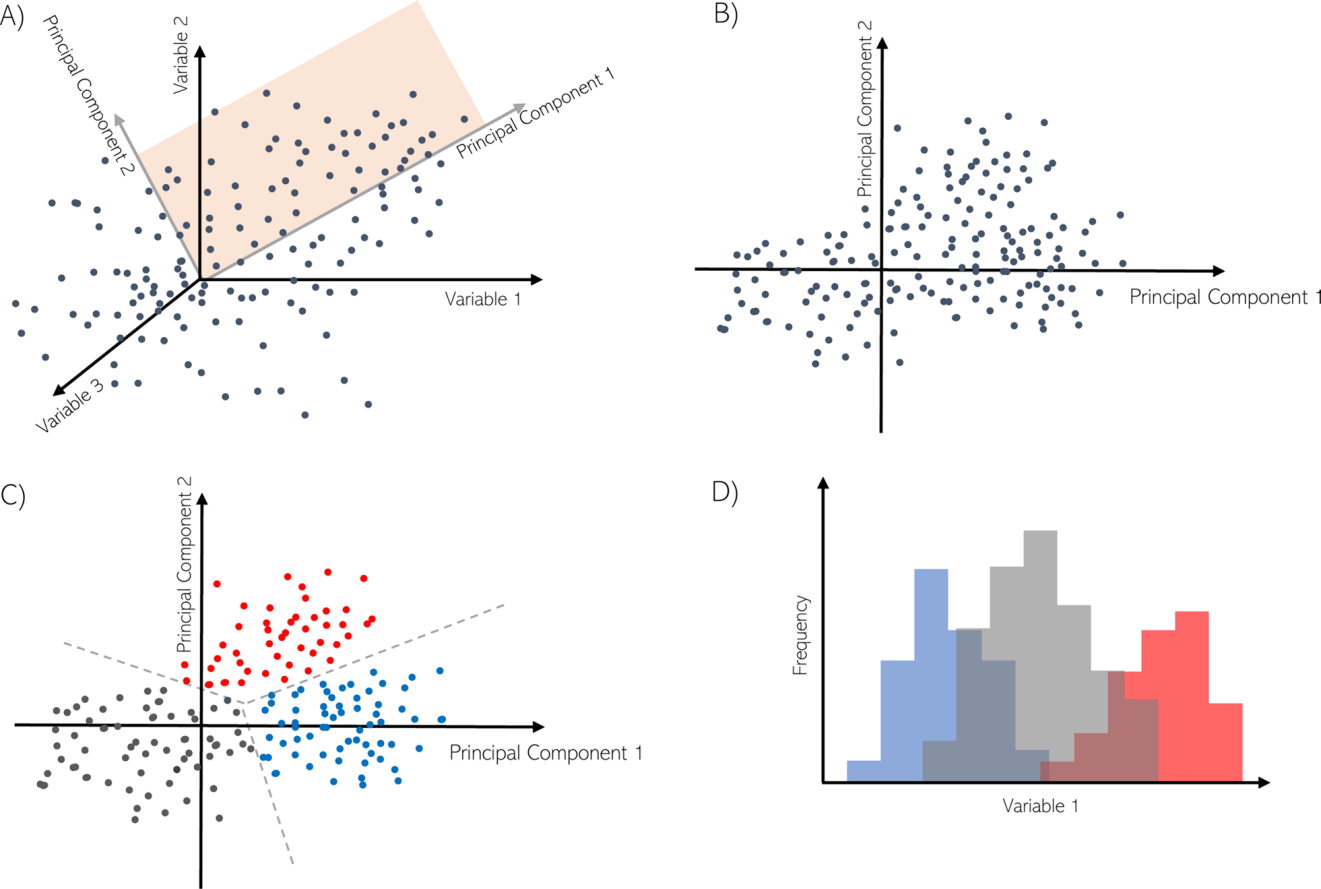
Methods for the data analysis. **A**) The data are expressed in terms of multiple relevant variables. **B**) A principal component analysis is performed to determine an underlying linear combination of the variables in order to maximize variability in terms of two “Principal Components”. **C**) A K-means analysis allows to split the data in a user-defined number of internally coherent clusters. **D**) The data, now expressed in terms of univariate distributions, is studied now in terms of the original variables and the recently arranged clusters

The dataset of ROIs described by end-expiratory aeration, tidal recruitment, and volumetric strain during both early and late stages (6 variables per ROI in total) considering all subjects was further analyzed using a clustering technique. To this end, ROI variables are first normalized so as to have zero mean and unitary variance. This normalized ROI dataset is then projected into a two-dimensional space using principal component analysis (PCA) using the scikit-learn library for Python [14]. In brief, PCA is a machine-learning technique used to reduce the dimensionality of a dataset. The principal components are linear combinations of the original variables which are computed upon the restriction that together they maximize the dataset variance while being orthogonal to the previous components (Fig. 1A). A K-Means clustering algorithm, implement in the scikit-learn library [14] was applied to the dataset of principal components to generate internally coherent clusters. K-means delivers groups of ROIs that are clustered together in the space of principal components using a distance criterion in reduced dimensional space. In our analysis, we selected three clusters according to the elbow method to be determined from the ROI dataset. As a result, each ROI receives a label related to the cluster it belongs to.

### Statistical analysis

To compare intra-group data at the beginning and at the end of the experiment, we used Wilcoxon signed rank test. Kruskal–Wallis H test, with Dunn’s test, were used for intra-group comparisons. The IBM SPSS software package (V.20.0; SPSS, Chicago, Illinois, USA) and GraphPad Prism V.5.0 (GraphPad Software, La Jolla, California, USA) were used for the statistical analyses. Data were expressed as the median ± IQR. Significance was set at *p* < 0.05.

## Results

The mean weight of the pigs was 27 ± 2 kg. All animals completed the experimental protocol, but in two subjects it was necessary to advance the acquisition of images due to terminal condition (at 8 and 9 h of VILI, respectively). The exclusion of images from these two subjects was necessary due to the severe damage to their lungs, which compromised the registration process and made pattern recognition between inspiration and expiration unreliable.

The animals developed an impairment in PaO_2_, Pplat, ΔP, respiratory system compliance, and central venous saturation in late stage compared with early stage (all *p* < 0.05), without significant changes in EVLW, GEDV, mean arterial pressure and arterial lactate. Also developed a loss of aeration at the end-of-expiration and end-of-inspiration, a decreased EELV, and an increased volumetric strain in the late stage (all *p* < 0.05) (Table S1, Online Supplementary Material).

### Cluster analysis

**Fig. 2:**
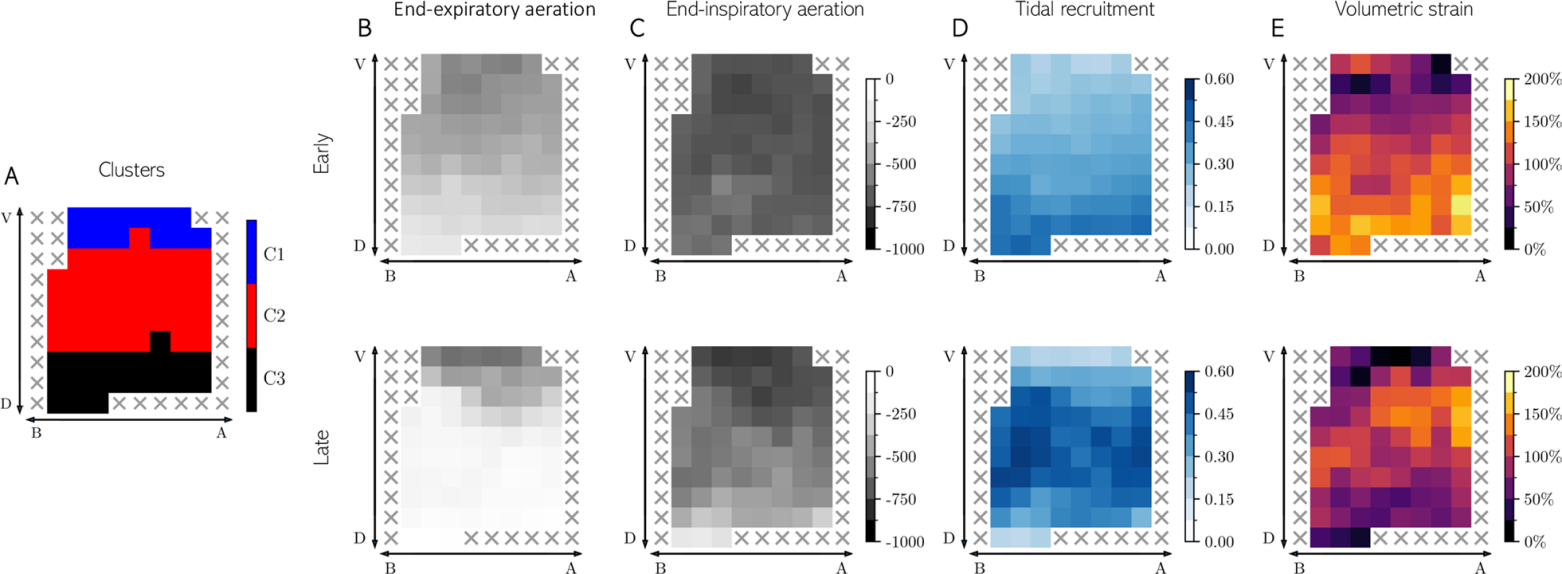
**A**) Distribution of ROIs according with clusterization: stable ROIs (blue), recruitable ROIs (red) and non-recruitable ROIs (black). Distribution of Hounsfield units (HU) in whole-lung computed tomography obtained at the **B**) end-of-expiration, and **C**) end-of-inspiration from early and late stages in the different clusters of lung parenchyma. **D**) Distribution of tidal recruitment according to the lung parenchyma clusters for the early and late stages. **E**) Distribution of volumetric strain according to the lung parenchyma clusters for the early and late stages for each of the lung parenchyma clusters

We identified three spatiotemporal clusters of lung tissue: C1 (17.7% of ROIs), C2 (45.1% of ROIs) and C3 (37.2% of ROIs). The comparative analysis of each cluster allowed us to test out the capacity of clusterization to effectively separate ROIs by their own characteristics, and in consequence to confirm that at early stage, all clusters were significantly different regarding end-expiratory aeration, tidal recruitment, and volumetric strain (Table 1). At late stage, we found a loss of end-expiratory aeration among C2 and C3 cluster, remaining unchanged in the C1 cluster (Fig. 2A). End-inspiratory aeration decreased in both the C3 and C2 clusters, remaining unchanged in the C1 cluster (Fig. 2B). Tidal recruitment slightly increased in the C1 and strongly increased in the C2, while decreased in the C3 (Fig. 2C). At the early stage, volumetric strain slightly increased in C2, while strongly decreased in C3, remaining unchanged in the C1 (Fig. 2D).

**Table 1:** Mean values for each cluster in terms of regional variables in the early and late stages

	Cluster size	Stable	Recruitable	Non-recruitable
		278	583	707
End-expiratory aeration [HU]	[E]	− 535 ± 169^†^	− 451 ± 135*^†^	− 279 ± 136*
	[L]	− 516 ± 247^†^	− 212 ± 274*^†‡^	− 85 ± 150*^‡^
End-inspiratory aeration [HU]	[E]	− 740 ± 149^†^	− 723 ± 89^†^	− 647 ± 126*
	[L]	− 747 ± 161^†^	− 612 ± 158*^†‡^	− 422 ± 163*^‡^
Tidal recruitment [–]	[E]	0.200 ± 0.079^†^	0.274 ± 0.076*^†^	0.360 ± 0.100*
	[L]	0.215 ± 0.081^†‡^	0.378 ± 0.124*^†‡^	0.292 ± 0.113*^‡^
Volumetric strain [%]	[E]	57.6 ± 41.7^†^	92.5 ± 44.4*^†^	99.1 ± 55.3*
	[L]	56.0 ± 53.7^†^	101.5 ± 47.5*^†‡^	44.6 ± 32.2*^‡^

Results expressed as median ± IQR

Intergroup analysis:

*Significant difference compared with Stable cluster

^†^Significant difference compared with Non-Recruitable cluster

Time-dependent analysis:

^‡^Significant changes were detected between early and late stage in any of the groups

In the following, we call a cluster “Stable” if the average ROI regional strain of the cluster ROIs does not significantly change from Early to Late stage, otherwise we considered the cluster to be “Unstable”. Further, we consider a cluster to be “Unstable-Recruitable” if average Tidal Recruitment significantly increases from Early to Late stages. Otherwise, we consider that cluster to be “Unstable-Non-Recruitable”.

**Fig. 3:**
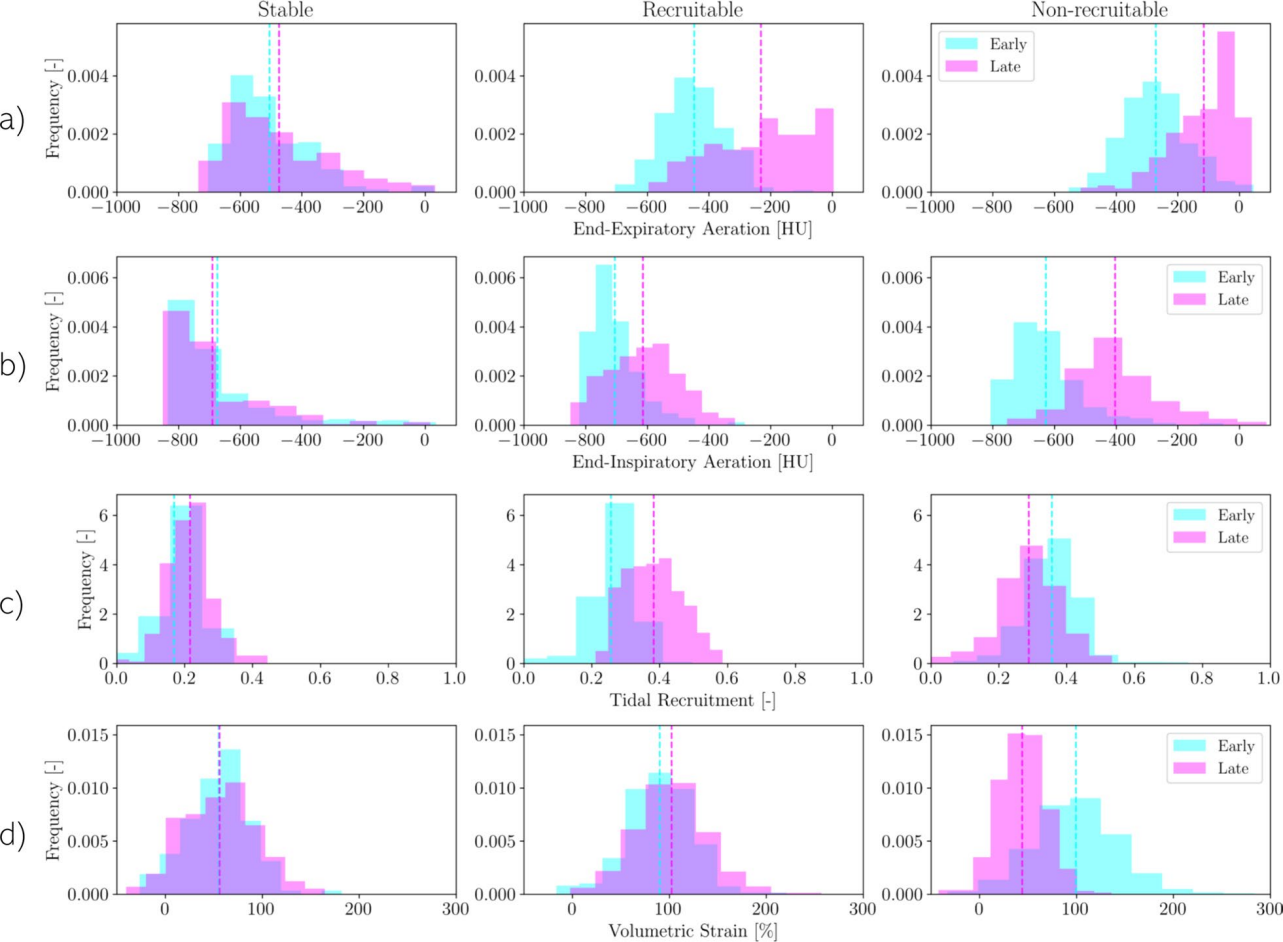
Topographic distribution of lung parenchyma clusters, regional end-expiratory and end-inspiratory aeration, tidal recruitment, and volumetric strain maps for representative subjects at early (top row) and late (bottom row) stages. It is shown how the clusters were distributed gravitationally: Stable Cluster (C1) located in the non-dependent zone, Recruitable Cluster (C2) in intermediate zones and Non-Recruitable Cluster (C3) in dependent zones. In the early and late stages Stable Cluster represents the best-preserved regions, where Tidal Recruitment and Volumetric Strain remains nearly unchanged. In contrast, Non-Recruitable Cluster had a at early stage, the least EE aerated, and the most tidal recruitment and volumetric strain, but with temporal decrease in tidal recruitment and volumetric strain. In the late stage, tidal recruitment and volumetric strain increased markedly in the Recruitable Cluster, suggesting that these regions mostly supplied the ventilation lost in the Non-Recruitable Cluster

Regarding the topographic distribution of the clusters, the Stable, Unstable-Recruitable, and Unstable-Non-Recruitable clusters were found predominantly in the ventral, intermediate, and dorsal compartments of the lung, respectively**.** The ROI analysis for a representative subject is included in Fig. 3.

## Discussion

The main finding of this exploratory 12-h MV experimental protocol, which employed high tidal volumes, was the spatial and temporal identification and classification of dynamic lung regions. This classification was based on parameters such as end-expiratory aeration, tidal recruitment, and strain, all measured at the protocol's initiation and conclusion. Remarkably, this approach naturally and unbiased proposes a functional categorization of lung damage, thereby aligning with gravity-dependent damage models previously proposed in scientific literature.

We implemented a one-hit acute lung injury model deliberately to ensure that the identified biomechanical phenomena could be directly attributed to the effects of high-stretch ventilation, eliminating potential interference from external factors such as saline solution lavages, acid aspiration, or endotoxin exposure. It is noteworthy that employing a model like this enabled us to replicate several consequences of injurious mechanical ventilation quite effectively. However, as with any model, we inevitably overlooked various other characteristics typical of ARDS lungs, such as inflammation, heterogeneity, and endothelial injury. These factors engage in a complex interplay that likely contributes to the progression or resolution of ARDS.

Protti et al. describe a highly effective VILI protocol using similar high Vt, around 40 ml/kg, without PEEP [3]. In their experiments, they observed widespread lung parenchymal damage and often encountered intense and rapid alveolar flooding, even before the first 12 h of the study. This phenomenon is known as ventilation-induced lung edema, and it is considered a proxy for regional lung damage, associated with both lung and systemic inflammation. Protti et al. further suggested that the flooding of airspaces resulted in collapse, reducing the regions available for ventilation, increasing global strain (consistent Vt but decreased functional residual capacity), and creating inhomogeneities within the lung parenchyma considered as stress raisers. Considering these observations, we intentionally reduced the Vt to 30 ml/kg to induce a less aggressive progression of lung injury. Despite this adjustment, we observed a substantial and often abrupt deterioration in lung function, characterized by worsened lung mechanics, hypoxemia, and the development of lung collapse. Furthermore, there is strong experimental evidence that regional collapse is a surrogate for regional damage in VILI [15].

The regionalization of strain through biomechanical analysis combined with K-Means Clustering analysis allowed us to identify functional regions that do not necessarily make up anatomical regions traditionally used in the evaluation of the lung parenchyma as unstructured regions of the lung. Clustering analysis constitutes a novel approach in the radiological study of lung pathophysiology, which allows to evaluate dimensionality from a large set of variables of regional biomechanical phenomena in an unbiased manner, allowing to identify patterns of mechanical behavior and, in this way, to cluster lung parenchyma areas according to multivariate encoding [15]. We carry out a final analysis by assessing the original variables under the previous clusterization. The found classification allows to discriminate patterns of behavior that help shedding light into the mechanisms underlying VILI.

The PCA of our biomechanical image-based assessment showed that different lung regions, which may reach similar levels of regional strain both in early and late stages frequently differ in their end-expiratory aeration. This finding contrasts with the notion of VILI purely based on regional strain as the sole predictor of damage, as it demonstrates that it is possible to have two compartments with similar regional strain values but different functional outcomes (e.g., non-recruitable vs. stable). This suggests that the functional evolution of lung tissue and its risk of damage cannot be solely predicted by the regional strain attributable to tidal volume. Other factors such as regional compliance, gravitational distribution of pulmonary flow, and static strain may explain this variability in results among different compartments.

We successfully identified three distinct functional lung compartments based on their biomechanical and ventilatory features. These compartments exhibited varying behavior in terms of aeration and strain, both spatially and temporally. These findings could have implications for understanding the temporal tomographic progression of ARDS [16], particularly in patients with COVID-19-associated ARDS (C-ARDS). Among C-ARDS patients, there is a notable evolution in lung volume, density, and the location of pulmonary opacities on CT scans over time. The progression of CT appearance follows a specific pattern that varies with the severity of the disease [17–19].

Despite the high degree of global strain and lung edema progression observed during this protocol, we identified a cluster of lung tissue that remains stable over time, i.e., a compartment where strain did not progress in time and stayed aerated without collapsing. Borges et al. showed that these normally aerated units were present predominantly in the ventral lung region. In addition, this region presented an important tidal increment of hyperinflated units using high tidal volumes. Remarkably, these lung regions presented a lower degree of inflammation assessed by uptake of ^18^F-FDG. A possible explanation for this phenomenon is static strain being higher than dynamic strain, as the ventral lung regions remain open during the entire respiratory cycle [16]. More recently, Protti et al. in a similar experimental setup than the article commented above, they compared the effects of different static and dynamic strain (reaching the same global strain) over the development of lung and systemic inflammation. They showed that the most important determinant for lung and systemic injury was the dynamic strain [20]. We conducted statistical tests to assess the hypothesis of differences between the examined clusters. However, given the presence of multiple ROIs per subject and cluster, a high level of data dependence is anticipated, by this reason clustering appears as an alternative which facilitates data exploration and the formulation of predictor variables, laying the groundwork for hypothesis testing in future studies based on these predictors.

The other lung compartments showed a more predictable behavior, aligned with what is expected during VILI. Regional strain significantly progressed in the Recruitable-unstable tissue but did result in decrease in the Non-recruitable-unstable tissue. We note that in the recruitable tissue, global strain increased in roughly 15% from early to late, in absolute terms. In this way, a migration of the damage pattern occurs between early and late stages. This observation is supported by the fact that the progression to lung collapse is higher in the Non-recruitable than in the Recruitable tissue. Since collapsed tissue is not expected to deform, it is the Recruitable tissue the one expected to deform the most, which is suggested by our regional strain analysis. In this way, global/regional strain is not able by itself to predict the future development of lung damage, as it is in previous studies. This highlights the complexity of the interaction of regional phenomena in the future development of VILI or the ability to predict it with the use of tomographic images. When the strain–stress threshold was overcome (Vt 38 ml/kg and global strain > 1.5), the consequences were devastating, developing severe pulmonary edema [3]. This situation occurs in the Non-Recruitable Unstable cluster identified in our work, while the Stable and Recruitable clusters are comparable with the non-Ventilator-induced lung edema-group (Vt 22 ml/kg and global Strain 1.29) [3]. When large strains are applied to the pulmonary extracellular matrix, the blood–gas barrier get locally deformed, and stress fatigue [20], rupture of microcapillaries [21] and inflammation [22] may occur, which converge in an increased permeability of lung capillaries, edema formation, lung mechanics and gas exchange impairment. However, not all aspects of lung injury can be attributed solely to the stretching of the alveolocapillary barrier; blood flow derangement secondary to oscillations in right ventricle preload and an increase in transmural left ventricular end-diastolic pressure also play significant roles [23].

Our study has several limitations, which we discuss next. First, our biomechanical analysis did not assess intermediate time instants between early and late stages. Given the high levels of injury at the late stage, it is hard to estimate when during the protocol regional inflammation started to occur, an aspect that future studies could elucidate. Second, the MV strategy used was highly injurious, and may not necessarily trigger a similar clusterization of regional strain and aeration in the context of protective MV. Third, our work did not study other important factors in VILI, such as vascular flow or regional inflammation, which could be synergic with regional strain and aeration. Fourth, this model was built using images from the very early (healthy lung) and the late stages of the VILI model. It is unclear how the model would behave when using CT images of already injured lungs. Despite these limitations, our study provides one of the first detailed accounts of spatiotemporal changes in regional parenchyma behavior in lungs subject to global strain levels that are known to induce VILI. Further, the novel use of machine-learning tools for the spatiotemporal analysis, and in particular clustering analysis, represents a proof of concept that such tools may confirm and enhance our understanding of regional mechanisms of VILI.

Looking forward, our biomechanical methodological approach utilizing CT images has the potential in predicting which lung regions are susceptible to injury progression during mechanical ventilation. To achieve this, validation in an independent cohort is essential.

## Conclusions

In summary, our study contributes a detailed and unbiased spatiotemporal analysis of dynamic lung regions in the context of VILI, shedding light on the complex interplay of different biomechanical factors. The biomechanical methodological approach, coupled with machine-learning tools, holds promise for predicting lung regions susceptible to injury progression during mechanical ventilation, opening new horizons for understanding and managing VILI. Validating this methodology in a new, independent cohort is essential to generalize these concepts.

### Supplementary Information

**Additional file 1: Table S1.** Physiological and imaging measurement data at early and late stage of injurious mechanical ventilation. Results expressed as median (IQR).

## Data Availability

The datasets used and analyzed during the current study are available from the corresponding author on reasonable request.

## Abbreviations

ARDS: Acute respiratory distress syndrome
C_RS_: Respiratory system compliance
CT: Computed tomography
EE: End-of-expiration
EI: End-of-inspiration
EVLW: Extravascular lung water
GEDV: Global end-diastolic volume
HU: Hounsfield unit
MV: Mechanical ventilation
PCA: Principal component analysis
PEEP: Positive end-expiratory pressure
Pplat: Plateau pressure
ROI: Region of interest
RR: Respiratory rate
SpO_2_: Arterial oxygen saturation
ScvO_2_: Central venous hemoglobin oxygen saturation
VILI: Ventilator-induced lung injury
Vt: Tidal volume
ZEEP: Zero end-expiratory pressure
