# Supplementary material for: A machine-learning regional clustering approach to understand ventilator-induced lung injury: a proof-of-concept experimental study
Source: Intensive Care Med Exp. 2024 Jul 2;12:60. doi: 10.1186/s40635-024-00641-8 (PMC11220131; doi:10.1186/s40635-024-00641-8)
Supplement: Supplementary file 1 — Additional file 1: Table S1. Physiological and imaging measurement data at early and late stage of injurious mechanical ventilation. Results expressed as median (IQR). [file 40635_2024_641_MOESM1_ESM.docx]

**Table S1.** Physiological and imaging measurement data at early and late stage of injurious mechanical ventilation. Results expressed as median (IQR).

|  | **Early Stage** | **Late Stage** | **P-value** |
| --- | --- | --- | --- |
|  |  |  |  |
| **Respiratory measurements** |  |  |  |
| PaO_2_ [mmHg] | 481 (29) | 61 (16) * | <0.01 |
| PaCO_2_ [mmHg] | 41 (9) | 38 (19) | 0.57 |
| Tidal volume [mL] | 796 (47) | 782 (78) | 0.29 |
| Plateau pressure [cmH2O] | 28.0 (3.0) | 40.0 (3.5) * | 0.02 |
| Driving pressure [cmH2O] | 26.9 (3.6) | 38.4 (3.9) * | 0.02 |
| Total PEEP [cmH2O] | 1.1 (0.4) | 1.4 (0.2) | 0.22 |
| Respiratory system compliance [ml/cmH2O] | 26 (5) | 20 (4) * | 0.03 |
|  |  |  |  |
| **Hemodynamic measurements** |  |  |  |
| Mean arterial pressure [mmHg] | 93 (43) | 83 (16) | 0.58 |
| Cardiac output [L/min] | 4.2 (0.6) | 4.8 (3.1) | 0.25 |
| Extravascular lung water [mL] | 283 (37) | 391 (76) | 0.12 |
| Global end-diastolic volume [mL] | 475 (102) | 452 (119) | 1 |
| Arterial lactate [mmol/L] | 0.8 (0.2) | 0.6 (0.5) | 0.09 |
| Central venous saturation [%] | 90 (7) | 66 (7) * | 0.02 |
| Hemoglobin [g/dL] | 7.3 (0.7) | 7.7 (1.3) | 0.72 |
|  |  |  |  |
| **HU compartments at End-of-Expiration** |  |  |  |
| Non-Aerated Tissue [%] | 9.3 (4.4) | 48.1 (29.5) * | <0.01 |
| Poorly-Aerated Tissue [%] | 50.9 (6.3) | 32.4 (14.4) * | <0.01 |
| Normally-Aerated Tissue [%] | 40.0 (14.6) | 16.6 (16.2) * | 0.04 |
| Hyperinflated Tissue [%] | 0.3 (0.2) | 0.3 (0.1) | 0.84 |
|  |  |  |  |
| **HU compartments at End-of-Inspiration** |  |  |  |
| Non-Aerated Tissue [%] | 2.6 (1.1) | 8.8 (3.2) * | <0.01 |
| Poorly-Aerated Tissue [%] | 11.5 (4.2) | 25.4 (14.2) * | 0.02 |
| Normally-Aerated Tissue [%] | 84.5 (4.6) | 62.7 (14.3) * | <0.01 |
| Hyperinflated Tissue, [%] | 0.8 (0.2) | 1.2 (0.4) * | <0.01 |
|  |  |  |  |
| **Global Biomechanical Analysis** |  |  |  |
| End-Expiratory Lung Volume [mL] | 730 (163) | 539 (318) | <0.01 |
| End-Inspiratory Lung Volume [mL] | 1540 (233) | 1509 (232) | 0.25 |
| Tidal recruitment [-] | 0.27 (0.07) | 0.31 (0.07) | 0.14 |
| Volumetric Strain [%] | 1.06 (0.26) | 1.89 (2.01) | <0.01 |
